# G Protein-Coupled Estrogen Receptor 1 (GPER1) Mediates Aldosterone-Induced Endothelial Inflammation in a Mineralocorticoid Receptor-Independent Manner

**DOI:** 10.1155/2021/5575927

**Published:** 2021-06-18

**Authors:** Ziwei Tang, Qifu Li, Qingfeng Cheng, Mei Mei, Ying Song, Zhipeng Du, Wenwen He, Jinbo Hu, Shumin Yang, Zhihong Wang

**Affiliations:** Department of Endocrinology, The First Affiliated Hospital of Chongqing Medical University, Chongqing 400044, China

## Abstract

**Objective:**

It has been increasingly appreciated that G protein-coupled estrogen receptor 1 (GPER1) mediates both proinflammatory and anti-inflammatory response of estrogen. It is also involved in some rapid vascular effects of aldosterone in a mineralocorticoid receptor (MR) independent manner. However, whether GPER1 mediates aldosterone-induced inflammation response in endothelial cells and its relationship with MR are yet undetermined and therefore require further explanation.

**Method:**

Based on the hypothesis that GPER1 plays a role in the aldosterone-related vascular inflammation, the present study utilized a model of human umbilical vein endothelial cells transfected with MR siRNA and induced for inflammatory response with increasing concentration of aldosterone.

**Results:**

It was discovered that induction of aldosterone had no effect on the expression of GPER1 but promoted the expression of MR. Suppression of MR did not influence GPER1 expression, and GPER1 was capable of mediating part of aldosterone-induced endothelial inflammatory response. This effect may involve phosphoinositide 3-kinases (PI3K) pathway signaling.

**Conclusion:**

These findings not only demonstrated the role of GPER1 in aldosterone-induced vascular inflammation but also suggested an alternative for pharmaceutical treatment of hyperaldosteronism considering the unsatisfying effect on cardiovascular risks with MR antagonists.

## 1. Introduction

Accumulated evidence has demonstrated the role of aldosterone in vascular endothelial inflammation [1–3]. It is appreciated that binding and translocation of cytosolic mineralocorticoid receptor (MR) by aldosterone will lead to activation of reactive oxygen species (ROS) as well as mitogen-activated protein kinase (MAPK)/nuclear factor kappa B (NF-*κ*B) signaling pathways [4, 5]. This MR dependent pathway was widely accepted as the genomic effect of aldosterone. However, decades of effort have shown that induction of aldosterone can also provoke rapid vascular response in an MR independent manner [6, 7]. This phenomenon sheds light on the role of non-MR receptors in the physiological regulation of aldosterone.

Among several potential membrane receptors, G protein-coupled estrogen receptor 1 (GPER1) has been proposed as the candidate of the nongenomic receptor of aldosterone, considering its comparably high affinity with the reaction strength being tenfold of MR [8]. The original ligand of GPER1 was estrogen. However, previous literature suggested that GPER1 was required for aldosterone-induced calcium influx and sodium/bicarbonate cotransporters activation as well as sodium proton exchange [9–11] in several nonvascular cell models. Besides, Gros et al. [12] demonstrated that GPER1 contributed to the rapid activation of extracellular signal-regulated kinase (ERK) and apoptosis of aldosterone-induced vascular smooth muscle cells. Though it is still controversial about the direct binding site with aldosterone, GPER1 has been proved to be involved in increasing activities of this steroid hormone. Yet, whether GPER1 contributes to the aldosterone-induced vascular inflammatory response is still unknown.

In models with estrogen being the ligand, the role of GPER1 in the regulation of inflammation was controversial. Studies of metabolic syndrome in GPER knockout mice revealed a higher level of plasma proinflammatory cytokines compared to wild type mice [13]. Jacenik et al. [14] also demonstrated the effect of GPER1 in lowering C-reactive protein (CRP) levels in Crohn's disease. These together advocate the anti-inflammatory role of GPER1. Interestingly, Cai et al. [15] showed the opposite effect of GPER1 in estrogen induced inflammation regulation. Their study suggested that GPER1 provoked the expression of classic inflammation cytokines tumor necrosis factor *α* (TNF-*α*) and monocyte chemoattractant protein-1 (MCP-1) and induced monocytosis in systemic lupus erythematosus (SLE). Since the role of GPER1 in aldosterone-induced inflammation or its interaction with MR when stimulated by aldosterone has never been investigated before, in the present study, we investigated whether GPER1 contributed to the aldosterone-induced vascular inflammation.

## 2. Methods

### 2.1. Materials

Human umbilical vein endothelial cells (HUVEC) were purchased from Sigma-Aldrich Canada Ltd. (200-05N). Aldosterone (HY-113313) and G1 (HY-107216) were purchased from MCE, USA. G15 was purchased from GLPBIO, USA (GC16618). Human interleukin 1 beta (IL-1*β*) ELISA kit was purchased from KeyGen Biotech, China (KGEHC002b). The anti-NOD-, LRR-, and pyrin domain-containing protein 3 (NLRP-3) (ab210491); anti-IL-1*β* (ab2105); anti-GPER1 (ab39742); and anti-MR (ab2774) antibodies were purchased from Abcam, UK. The second antibodies were purchased from KeyGen Biotech, China. The PI3K inhibitor LY294002 (#9901) was purchased from Cell Signaling Technology, USA.

### 2.2. Cell Cultures and Treatment

HUVEC were cultured in 98% EGM-2 (Endothelial Cell Growth Medium-2), 2% FBS, and ECGS (Endothelial Cell Growth Supplement) at 37°C, 5% CO2. Passages between 3 and 4 were utilized for the experiment. Cells with or without control RNA (scRNA) or MR interfering RNA (siMR) were treated with PBS or aldosterone (from 10^−11^ M to 10^−9^ M) for 1 h to test inflammation factors changes. Another group of cells with transfected siMR were pretreated with PBS or aldosterone (from 10^−11^ M to 10^−9^ M) for 1 h and further treated with G15 (from 10^−10^ M to 10^−9^ M) for 1 h to assess whether GPER1 was involved in the inflammatory response. To understand the potential mechanism of GPER1 in inflammatory response, wild type cells were pretreated with saline or G1 (1 *μ*mol/L) for 1 h and then LY294002.

### 2.3. Generation of siMR and Cell Transfection

The siMR used in this experiment was generated by KeyGen Biotech, China, with the following sequence: 5′ to 3′ CAA GGA AGC AGC AAA GAA ATT, 3 ′to 5′ UUU CUU UGC UGC UUC CUU GTT. Before transfection, cells were seeded into 6-well multiple dishes to reach confluence between 70% and 80%. Dilute 2.5 *μ*g scRNA or siMR with 250 *μ*L Opti-MEM, mix them well, and incubate them at room temperature for 5 minutes. Dilute 5 *μ*L Lipo3000 with 250 *μ*L Opti-MEM, mix them well, and incubate them at room temperature for 5 minutes. Mix the diluted scRNA or siMR and Lipo3000, and incubate them at room temperature for 20 minutes. Add the scRNA/siMR-Lipo3000 mixture to 500 *μ*L cell culture suspensions, culture them for 6 h, and replace the medium. Then culture cells at 37°C, 5% CO2, for 48 h.

#### 2.3.1. Assessment of Endogenous Expression with Real-Time PCR

Isolation of total RNA was performed utilizing the one-step RNA isolation reagent TRIzol (Invitrogen, Carlsbad, CA). Measure the concentration and purity with NanoDrop, and make sure that the ratio of OD260 nm/OD280 nm is within 1.8–2.1. cDNA was generated using a commercial reverse transcription kit (TaKaRa, Japan, RR036B). Dilute cDNA by 10-fold, and make 20 *μ*L PCR system with 2x SYBR Green. Run with ABI StepOnePlus Real-Time PCR System. The primers' sequence was as follows: glyceraldehyde 3-phosphate dehydrogenase (GAPDH) F-CAAATTCCATGGCACCGTCA, R-AGCATCGCCCCACTTGATTT; intercellular adhesion molecule 1 (ICAM-1) F-AACCAGAGCCAGGAGACACT, R-GAGACCTCTGGCTTCGTCAG; vascular cell adhesion molecule 1 (VCAM-1) F-CCGTCTCATTGACTTGCAGC, R-GATGTGGTCCCCTCATTCGT; NLRP-3 F-AGAAGCTCTGGTTGGTCAGC, R-GTCTCCCAAGGCATTCTCCC; MR F-AGTGGAAGGGCAACACAACT, R-ACTTCTTTGACTTTCGTGCTCC.

#### 2.3.2. Assessment of Endogenous Expressions with Western Blot

Wash the cells with ice-cold PBS twice and digest them with trypsin at 37°C. Cells then were lysed with ice-cold lysis buffer (each 1 ml contained 10 *μ*L phosphatase inhibitor, 1 *μ*L protease inhibitor, and 5 *μ*L 100 mM PMSF). Shake at 4°C for 5 minutes and centrifuge with 14000 rpm at 4°C for another 5 minutes. Retrieve the upper layer and measure concentration using Bradford method. Resultant whole cell lysates were resolved by SDS-PAGE and transferred electrophoretically onto NC membranes. Then, membranes were blocked with 5% skim milk powder, washed with TBST, and blotted overnight with anti-NLRP-3 (0.5 *µ*g/ml), anti-IL-1*β* (2 *µ*g/ml), anti-GPER1 (4 *µ*g/ml), or anti-MR (5.6 *µ*g/ml) antibodies. Blots were washed in TBST and then incubated in anti-mouse (1 : 1,000 dilution) or anti-rabbit (1 : 5,000 dilution) antibodies. Proteins were visualized with G:BOX Chemi XR5 and analyzed with Gel-Pro32 software.

#### 2.3.3. Assessment of Secreted IL-1*β* with ELISA

Cell culture medium was diluted and measured for IL-1*β* level according to the manufacturer's protocols (KeyGen Biotech, China, KGEHC002b).

## 3. Statistical Analysis

Data were expressed as means ± SEM For two group comparisons, and Student's *t*-test was provided. For multiple group comparisons, initial analysis by one- or two-way ANOVA was followed by Student–Newman–Keuls post hoc test. *P* < 0.05 on a two-sided test was taken as a minimum level of significance.

## 4. Results

### 4.1. Inflammatory Response before and after MR Knockdown

**Figure 1:**
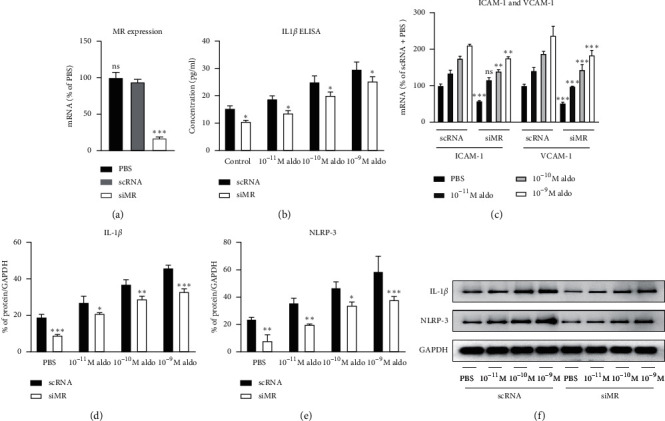
Inflammatory response before and after MR knockdown with different aldosterone concentrations. (a) MR mRNA expression; (b) level of cell culture medium IL-1*β*; (c) ICAM-1 and VCAM-1 mRNA expressions; (d) protein level of IL-1*β*; (e) protein level of NLRP-3; (f) Western blot of IL-1*β* and NLRP-3. scRNA: the state with control RNA, siMR: the state after MR knockdown, aldo: aldosterone. ^*∗*^*p* value < 0.05 compared between scRNA and siMR with the same aldosterone concentration, ^*∗∗*^*p* value < 0.01 compared between scRNA and siMR with the same aldosterone concentration, ^*∗∗∗*^*p* value < 0.001 compared between scRNA and siMR with the same aldosterone concentration. ns: nonsignificant.

Cells morphology was analyzed with microscope before and after transfection (Supplementary Material 1). After MR knockdown, the expression of MR was 83% (*p* < 0.001) less than control group without aldosterone stimulation (Figure 1(a)), suggesting successful siMR transfection. The protein level of IL-1*β* and NLRP-3 and the mRNA expressions of ICAM-1, as well as VCAM-1, along with the secreted IL-1*β*, were significantly decreased compared with the scRNA group with various aldosterone stimulation modes (Figures 1(b)–1(f)). However, the inflammatory response was not abolished in proportion with decrease of MR expression. The remaining inflammation was further strengthened in response to increasing aldosterone stimulation. This phenomenon suggested existence of a non-MR effect.

### 4.2. The Effect of MR Knockdown and Aldosterone Stimulation on GPER1 Expression

**Figure 2:**
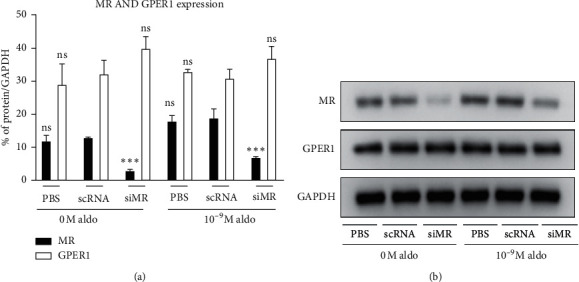
MR and GPER1 protein level before and after MR knockdown with or without aldosterone: (a) protein level of MR and GPER1; (b) Western blot of MR and GPER1. scRNA: the state with control RNA, siMR: the state after MR knockdown, aldo: aldosterone. ^*∗∗∗*^*p* value < 0.001 compared with scRNA under the same aldosterone concentration. ns: nonsignificant compared with scRNA under the same concentration.

Unlike MR expression, which positively responded to aldosterone stimulation, the GPER1 protein level was comparable before and after aldosterone stimulation. In addition, without aldosterone, the proteomic MR expression of siMR group decreased by 76.9% (3 ± 0.7% vs. 13 ± 0.5%, *p* < 0.001) compared with scRNA group, but the protein level of GPER1 showed no change (Figure 2). This phenomenon was also demonstrated even after 10^−9^ M aldosterone stimulation, indicating that GPER1 expression was not influenced by the change of MR regardless of the existence of aldosterone.

### 4.3. The Role of GPER1 in Aldosterone-Induced Inflammation

**Figure 3:**
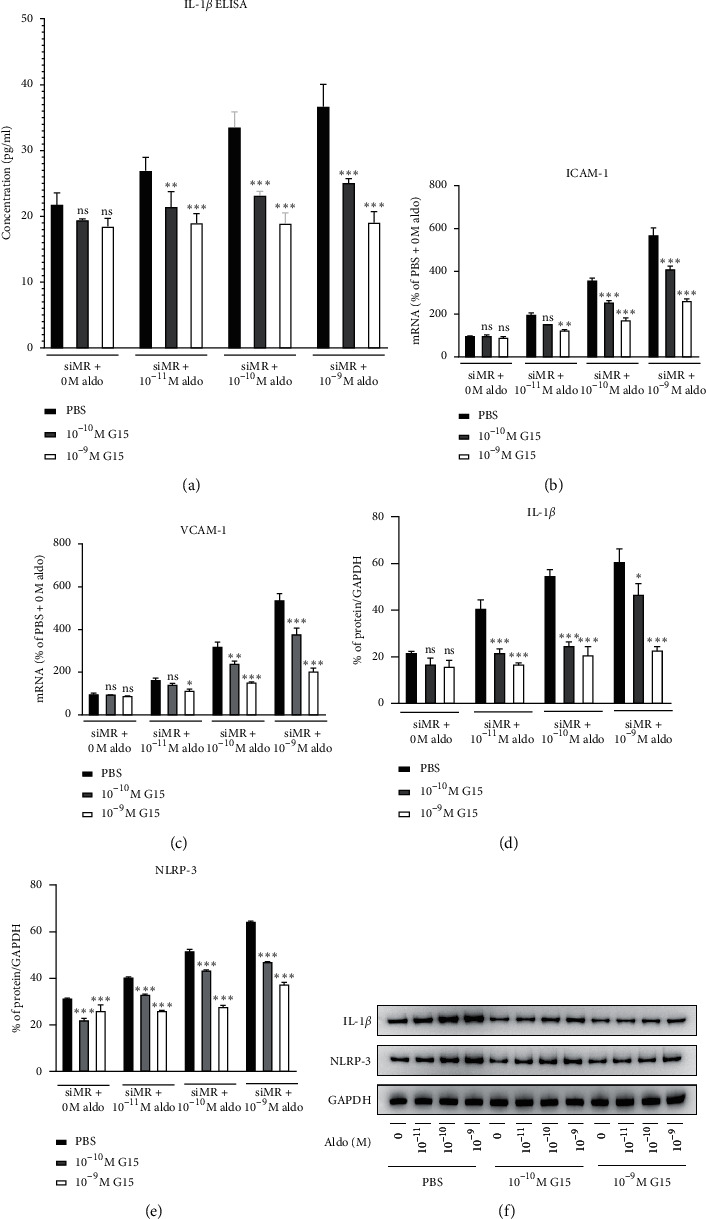
Inflammatory response before and after treatment with different concentrations of G15: (a) level of cell culture medium IL-1*β*; (b) ICAM-1 mRNA expression; (c) VCAM-1 mRNA expression; (d) protein level of IL-1*β* from PBS to 10^−9^ M G15; (e) protein level of NLRP-3 from PBS to 10^−9^ M G15; (f) Western blot of IL-1*β* and NLRP-3 from PBS to 10^−9^ M G15. SiMR: the state after MR knockdown, aldo: aldosterone. ^*∗*^*p* value < 0.05 compared with PBS within the same aldosterone concentration, ^*∗∗*^*p* value < 0.01 compared with PBS within the same aldosterone concentration, ^*∗∗∗*^*p* value < 0.001 compared with PBS within the same aldosterone concentration. ns: nonsignificant compared with PBS within the same aldosterone concentration.

To determine whether GPER1 would contribute to the inflammation following the change of aldosterone concentration, the transfected cells were treated with GPER1 specific antagonist G15. The results demonstrated that ELISA measurement of IL-1*β* and mRNA expressions of ICAM-1 and VCAM-1 (Figures 3(a)–3(c)), as well as protein expression of IL-1*β* and NLRP-3 (Figures 3(d)–3(f)), were significantly decreased after G15 treatment.

When the concentration of G15 increased from 10^−10^ M to 10^−9^ M, the inflammation response decreased to a further extent. For IL-1*β* from cell culture medium, when G15 concentration reached 10^−9^ M, the results were comparable between groups with different amount of aldosterone stimulation and had no difference compared with the one without aldosterone or G15 treatment. This phenomenon was also proved in the Western blot analysis of IL-1*β*, indicating maximum inhibition of GPER1.

### 4.4. Assessment of PI3K Pathway

**Figure 4:**
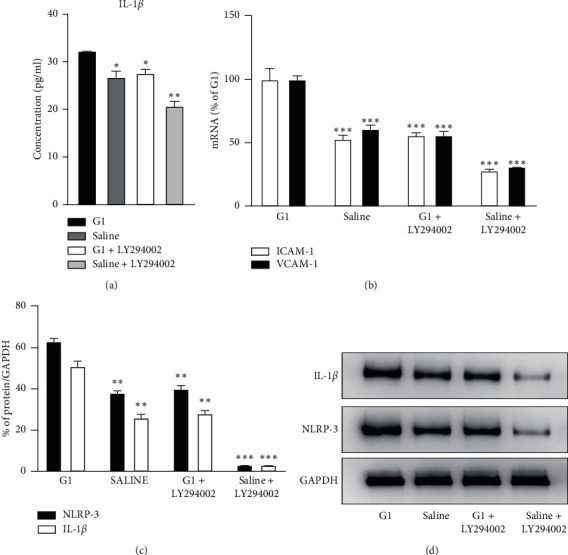
Inflammatory response before and after inhibition of the PI3K pathway: (a) level of cell culture medium IL-1*β*; (b) ICAM-1 and VCAM-1 mRNA expression; (c) protein level IL-1*β* and NLRP-3. (d) Western blot of IL-1*β* and NLRP-3. ^*∗∗*^*p* value < 0.01 compared with G1, ^*∗∗∗*^*p* value < 0.001 compared with G1.

To reduce the impact of MR on PI3K pathway, we applied GPER1 specific agonist G1 to stimulate HUVEC. With G1 induction, the endogenous level of NLRP-3, IL-1*β*, ICAM-1, and VCAM-1, as well as secreted IL-1*β*, was significantly increased (Figures 4(a)–4(d)). After application of PI3K inhibitor LY294002, the secreted IL-1*β* decreased by 14.37% (32.46 ± 0.18 pg/ml vs. 27.76 ± 1.02 pg/ml, *p* < 0.01), the protein expression of NLRP-3 and IL-1*β* reduced by 36.5% and 45.1% (*p* < 0.01), respectively, and the mRNA expression of ICAM-1 and VCAM-1 reduced by 44% (*p* < 0.01), all of which were significantly different from the G1 group.

## 5. Discussion

The role of GPER1 in the regulation of inflammation was controversial. In a model of atherosclerosis, Bowling et al. [16] once demonstrated that GPER1 was involved in the estrogen related anti-inflammatory effect on endothelial cells and macrophages. In different types of models, such as ischemic stroke [17], GPER1 on microglia was also suggested to mediate the effect of decreasing IL-1*β* and TNF-*α* under G1 or estradiol stimulation. On the contrary, Heublein et al. [18] found out that, in ovarian endometriosis and pelvic inflammatory disease models, the expression of GPER1 in ovaries was upregulated, suggesting the role of GPER1 in promoting ovarian inflammation. In the present study, the role of GPER1 in aldosterone-induced endothelial inflammation was investigated for the first time. According to our observation, when treated with aldosterone, the inflammatory response was not reduced in proportion with the knockdown of MR but significantly reduced after G15 treatment. Since G15 was described to have no obvious effect on MR's function [10], we believed that the inflammation reduction after G15 treatment was specifically contributed by GPER1. For protein analysis, when the concentration of G15 was increased to 10^−9^ M, the decrease in NLRP-3 and IL-1*β* was around 50%. For mRNA assessment of ICAM-1 and VCAM-1, when G15 reached 10^−9^ M, the increase of each factor after aldosterone induction (10^−11^ M and 10^−10^ M) was almost completely blocked to a level comparable with the group without treatment of aldosterone. These together suggested the central role of GPER1 in promoting aldosterone stimulated endothelial inflammation after inhibition of MR.

It is important to note that aldosterone may not have a direct binding site of GPER1, as demonstrated by previous research [11, 19]. Therefore, it is vital to provide evidence on the interaction between GPER1 and other receptors of aldosterone to determine whether the effect of GPER1 in aldosterone-induced inflammation was independent. In Rigiracciolo's work [11], GPER1 was involved in the cross-talk with MR in the aldosterone-induced proliferation of breast cancer cells. The change in GPER1 expression was synchronized with MR when stimulated by aldosterone. On the contrary, in Gros's work [10] with different cell model, transfection of MR downregulated GPER1 expression and vice versa. Our study also showed a different result. Without aldosterone stimulation, the protein level of GPER1 was not influenced by the knockdown of MR expression in HUVEC. After induction with 10^−9^ M aldosterone, the expression of GPER1 did not differ from that of 0 M aldosterone. The increase in MR after aldosterone stimulation also seemed to have no effect on GPER1 expression, suggesting that these two receptors might be independent of each other in this HUVEC model. It might partly explain the remaining cardiovascular risks after MR antagonist treatment in primary aldosteronism patients. The reason for the difference between our results and Rigiracciolo's work was still undetermined. We suggested that the expression pattern may vary in different cell models and related to different aldosterone concentrations. Considering the fact that inflammation response was increased following aldosterone induction, even though the expression of GPER1 was not upregulated and the inflammatory effect could be inhibited via G15 treatment, we suggested that the enhancement of GPER1 activity rather than the increase in expression might be responsible for this phenomenon. Therefore, further studies on the activity of GPER1 after aldosterone induction might be considered.

Pei et al. [20] once reported that GPER1 inhibits angiotensin II induced cardiomyocyte hypertrophy by upregulating the PI3K-Akt-mTOR signaling pathway. Moreover, in Jiang's work [21], GPER1 prevents the apoptosis of retinal ganglion cells mainly by the PI3K/Akt pathway. In a model of osteoarthritis [22], which is a progressive inflammation joint disease, the role of GPER1 in protecting chondrocytes against mitophagy was via PI3K signaling. In our present study, we also investigated the potential mechanism for GPER1 related inflammation. According to our observation, G1 could induce elevation of NLRP-3 and IL-1*β* as well as ICAM-1 and VCAM-1. This effect was believed to be GPER1 specific since De Giusti et al. once demonstrated no effect on MR or other estrogen receptors [10]. In our study, we suggested that GPER1 may induce inflammation response via the PI3K pathway. After the treatment of PI3K inhibitor, the secreted IL-1*β*, the protein expression of NLRP-3 and IL-1*β*, and the mRNA expression of ICAM-1 and VCAM-1 all significantly decreased, with a level comparable to the group treated with saline. It is the first time that the signaling of GPER1 in aldosterone-induced endothelial inflammation has been discussed, and the results are consistent with previous discoveries. Though GPER1 may induce endothelial inflammation via the PI3K pathway, we have to consider that there might also be another pathway such as MAPK signaling [23,24] since the inflammation response after treatment of PI3K inhibitor was not completely blocked. Future studies on other pathways might be required. The elementary schematic drawing of the mechanism is provided in Supplementary Material 2.

## 6. Conclusion

The expression of GPER1 and MR seemed to be independent in human umbilical vein endothelial cells with or without aldosterone stimulation. Knockdown of MR failed to proportionally decrease the inflammation response after aldosterone stimulation. Through application of GPER1 specific antagonist G15, it was proved that GPER1 mediates part of aldosterone-induced endothelial inflammatory response. This effect might involve the role of the PI3K pathway. Our findings indicated that GPER1 might have different roles in the regulation of inflammation when confronted with aldosterone compared with estrogen.

## Data Availability

The datasets generated and/or analyzed during the current study are available from the corresponding author upon reasonable request.

## Supplementary Materials

Supplementary Materials
*Supplementary Material 1*. Cell morphology under microscope before and after siMR transfection. Cells were treated with PBS, control RNA (scRNA), or MR interfering RNA (siMR). The cells were all alive during transfection process. *Supplementary Material 2*. Schematic drawing of the mechanism involving GPER1 and MR when HUVEC were stimulated with aldosterone. According to our research, GPER1 is involved in the aldosterone stimulated endothelial inflammation process, being independent of MR, probably through PI3K pathway, but what still remains unknown is how GPER1 interacts with aldosterone and PI3K.
