# Supplementary material for: G Protein-Coupled Estrogen Receptor 1 (GPER1) Mediates Aldosterone-Induced Endothelial Inflammation in a Mineralocorticoid Receptor-Independent Manner
Source: Int J Endocrinol. 2021 Jun 18;2021:5575927. doi: 10.1155/2021/5575927 (PMC8235990; doi:10.1155/2021/5575927)
Supplement: Supplementary Materials — Supplementary Material 1. Cell morphology under microscope before and after siMR transfection. Cells were treated with PBS, control RNA (scRNA), or MR interfering RNA (siMR). The cells were all alive during transfection process. Supplementary Material 2. Schematic drawing of the mechanism involving GPER1 and MR when HUVEC were stimulated with aldosterone. According to our research, GPER1 is involved in the aldosterone stimulated endothelial inflammation process, being independent of MR, probably through PI3K pathway, but what still remains unknown is how GPER1 interacts with aldosterone and PI3K. [file 5575927.f1.docx]

**20x**

| +PBS | +scRNA | +siMR |
| --- | --- | --- |
| 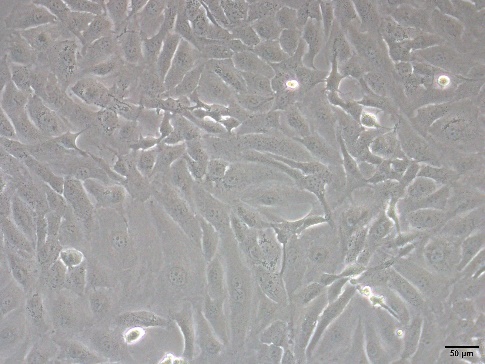 | 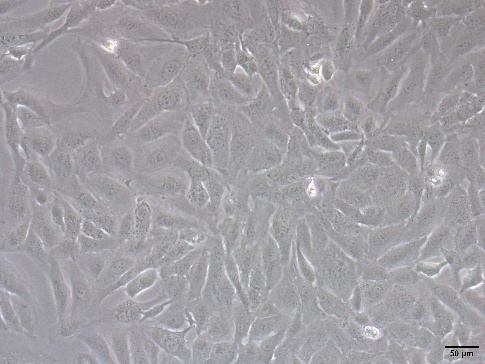 | 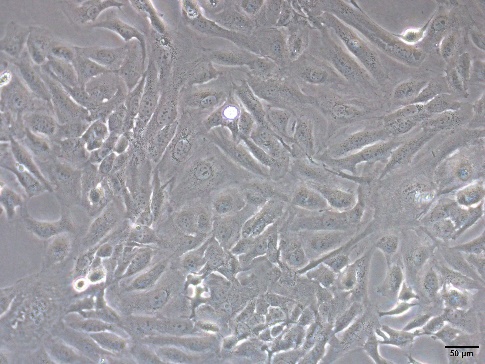 |

**40x**

| 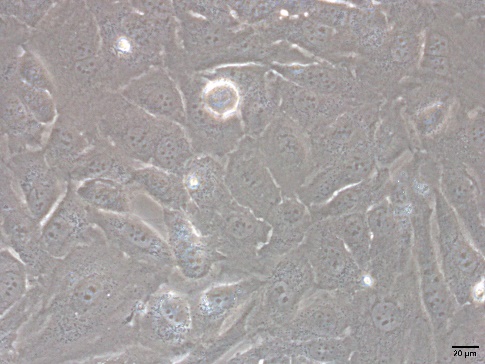 | 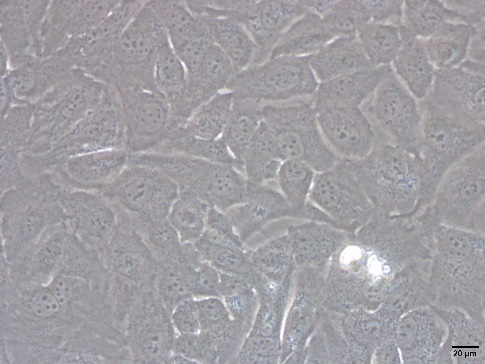 | 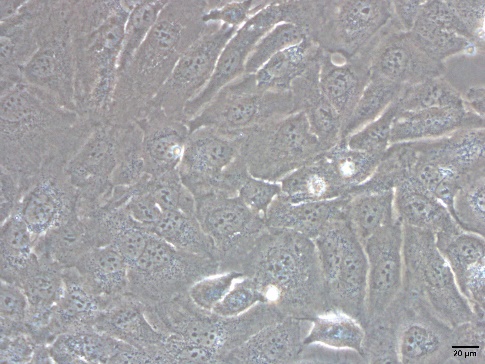 |
| --- | --- | --- |

Supplement 1. Cell morphology under microscope before and after siMR transfection. Cells were treated with PBS or control RNA(scRNA) or MR interfering RNA(siMR).


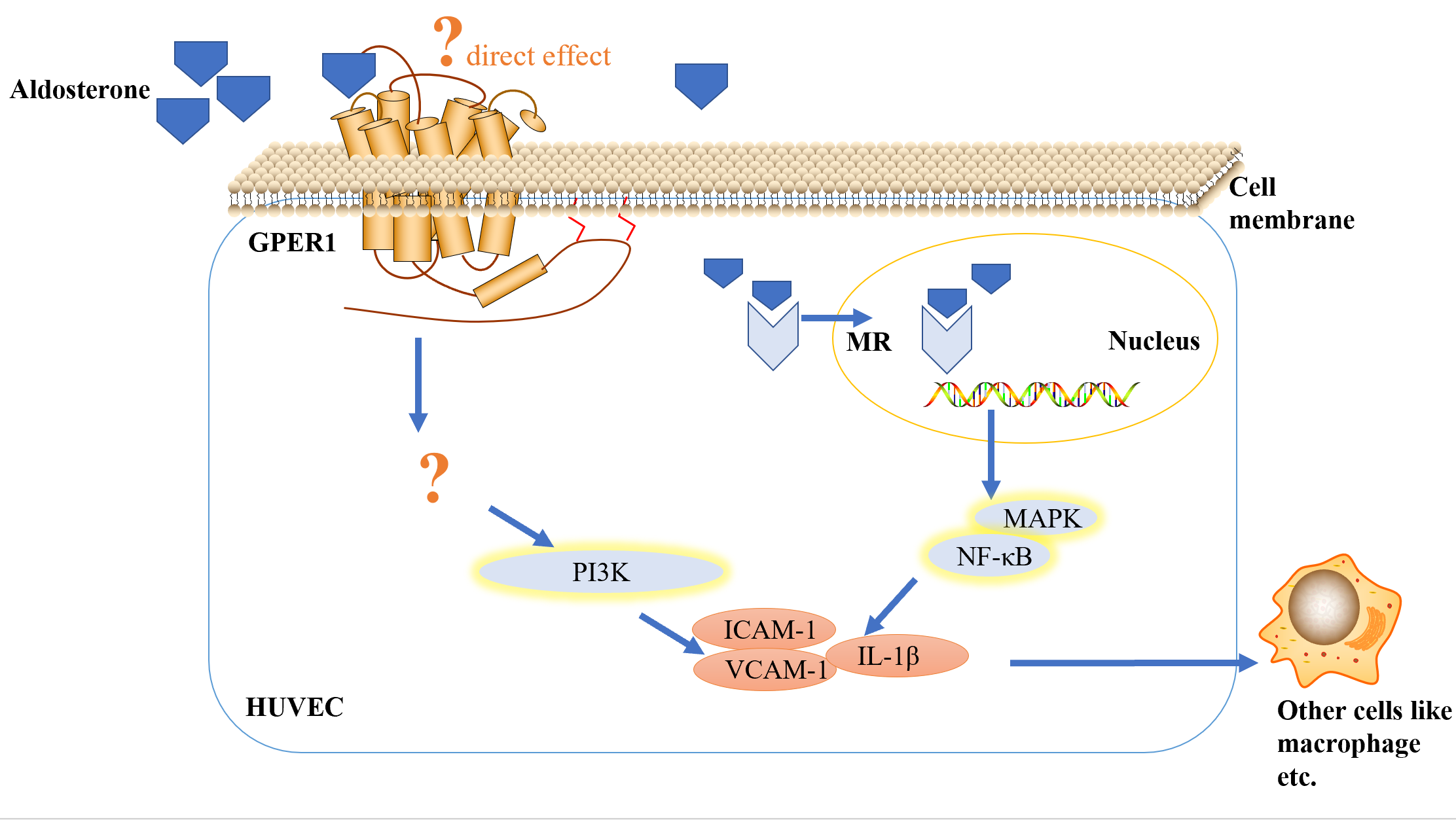


Supplement 2. Schematic drawing of the mechanism involving GPER1 and MR when HUVEC stimulated with aldosterone.
